# Translation, cross-cultural adaptation and validation of the universal Welch Emotional Connection Screen using primary and bilingual Spanish-speaking coders of videotaped mother-child interactions

**DOI:** 10.3389/frcha.2024.1346121

**Published:** 2024-08-26

**Authors:** Amie A. Hane, Robert J. Ludwig, Amy G. Martinez, Cynthia Masese, Ulla Vanhatalo, Cliff Goddard, Marc E. Jaffe, Michael M. Myers, Martha G. Welch

**Affiliations:** ^1^ Department of Psychology, Bronfman Science Center, Williams College, Williamstown, MA, United States; ^2^ Department of Pediatrics, Columbia University Irving Medical Center, New York, NY, United States; ^3^ Department of Languages, University of Helsinki, Helsinki, Finland; ^4^ School of Humanities, Languages and Social Science, Griffith University, Brisbane, QL, Australia; ^5^ Children’s Learning Centers of Fairfield County, Stamford, CT, United States; ^6^ Division of Developmental Neuroscience, New York State Psychiatric Institute, New York, NY, United States; ^7^ Department of Anatomy and Cell Biology, Columbia University Irving Medical Center, New York, NY, United States; ^8^ Department of Psychiatry, Columbia University Irving Medical Center, New York, NY, United States

**Keywords:** emotional behavior, screening tool, translatability, emotional connection, mother-child interaction, videotape assessment

## Abstract

**Introduction:**

Using clear explicit translatable language, we translated the Welch Emotional Connection Screen into a new universal language instrument, the *English uWECS*. In this study, we had two aims: Aim 1 was to establish *concurrent validity* of the uWECS by comparing scores coded by primary Spanish-speaking coders using the Spanish translation of the uWECS to scores coded by bilingual, secondary Spanish-speaking coders using the oWECS. Aim 2 was to establish the *criterion-related validity* in terms of oWECS and uWECS performance in tracking change in autonomic emotional connection (AEC) during the course of an intervention among preschool aged children.

**Methods:**

We created a library of 52 five-minute Spanish-speaking mother-child videos that were collected during a randomized controlled trial of Mother-Child Emotional Preparation intervention (MCEP). The videos were collected at two time points, at enrollment and at a 6-month follow-up. The subsample of Primary Spanish-Speaking dyads from the MCEP study were coded by two independent teams of coders. We trained primary English-speaking (bilingual Spanish) coders on the oWECS, using the original training program. A different team of primary Spanish-speaking coders coded the same cases using the novel uWECS guide and trained briefly for reliability with the Spanish uWECS translation materials.

**Results:**

We found that the Spanish oWECS and Spanish uWECS ratings from the baseline and 6-month follow-up observations were robustly correlated, with intraclass correlations ranging from .81 to .84 and all *p*-values<.001, thus demonstrating sound concurrent validity for the uWECS. The oWECS and uWECS scores also achieved parallel results when evaluating the efficacy of the MCEP for primary Spanish-speaking dyads. Both the AEC scores of the oWECS [*F*(1, 27) = 4.31, *p* < .05] and the scores of the uWECS [*F*(1,27) = 4.06, *p* < .05] similarly demonstrated significant change post intervention, thus demonstrating sound criterion-related validity of the uWECS.

**Discussion:**

These findings demonstrate that the uWECS can be used to measure parent/child AEC in linguistically diverse populations and cultures.

## Introduction

Research focusing exclusively on translation of observational methodologies for use by linguistically diverse populations is scarce. However, cross-cultural validation of observational methods is necessary to validate the construct being coded. Only if the instrument itself is readily translatable, is it possible to determine whether the measure is capturing the same central construct in a way that is applicable, meaningful, and relevant across cultures and languages (1). Some self-reported health status measures have been adapted for use in languages other than the source language (2). However, the cross-cultural adaptation process is also important when an instrument is used in a different language/setting/time to reduce the risk of introducing bias into a study (3). Research questionnaires are not always translated appropriately before they are used in new temporal, cultural or linguistic settings (4). The results based on such instruments may therefore not accurately reflect the central constructs they are designed to measure.

In recent years, efforts have been made to translate and adapt Western assessment tools that measure emotional constructs for use in diverse languages and cultures, e.g., tools that assess a child's psychological self-regulation of emotions, or the child's ability to identify and manage their emotions and feelings (5, 6). The same attention has not been paid to the translation of observational methodologies used to rate mutual emotional constructs between parents and young children. However, one recent study demonstrates the importance and challenges involved in doing so. Schneider and colleagues coded the quality of mother-child interaction to validate the Responsive Interaction for Learning (RILF) measure for use with Brazilian families (7). This study applied back-translation (English to Portuguese, and back again to English) before training primary or exclusively Portuguese-speaking coders to ensure the cultural and linguistic validity of the coding. This back-translation approach revealed that some terms did not clearly translate (e.g., “mind reading”) and scaling and descriptive anchors were not relevant as written (e.g., 3 items were reworded and scaling changed/extended). These issues were resolved by modifying the RILF, in order to make it relevant for Portuguese coders—resulting in a validated Portuguese version of the measure, the RILF-P (7). Importantly, the same labor-intensive procedure would need to be followed for adapting the RILF to other languages.

**Figure 1:**
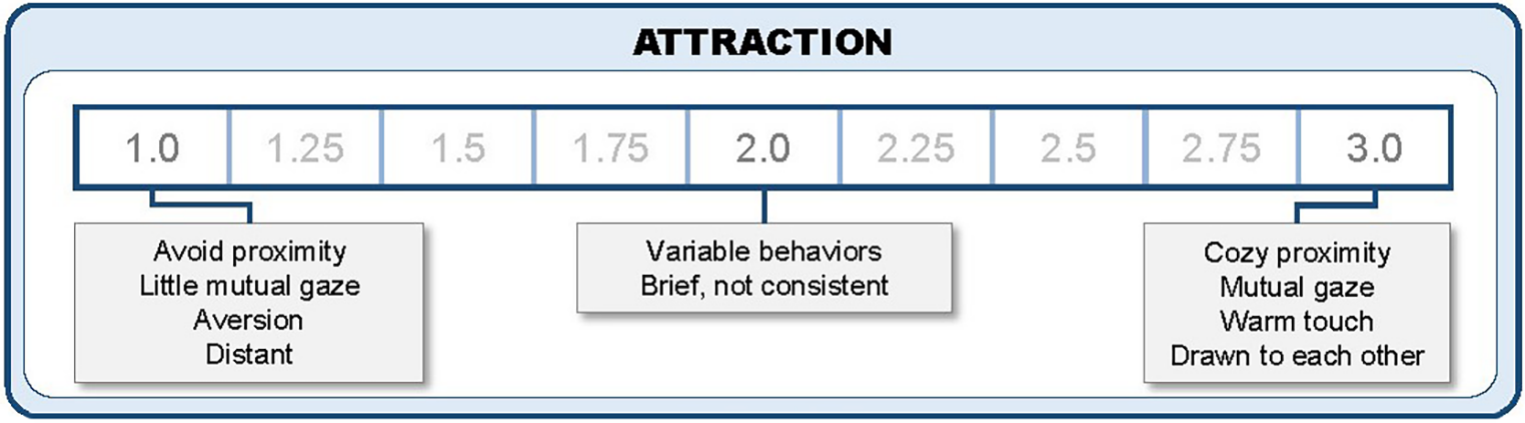
The original English version of the welch emotional connection screen (oWECS).

The uWECS overcomes this issue with o*ne core translation of the instrument*—i.e., translating the WECS into Clear, Explicit, Translatable Language (CETL); (8) to assess the behavioral indicators of autonomic emotional connection between mother and child. Autonomic emotional connection (AEC) is a construct that describes the emotional behaviors of mother and baby that are triggered by the autonomic nervous system. Based upon clinical observations of parents and children from diverse cultures speaking multiple languages, we created a novel assessment tool, the original English Welch Emotional Connection Screen (oWECS) to assess the AEC construct. The idea behind the oWECS was that the AEC construct itself was intuitive and common to all cultures and therefore language should not be a barrier. The primary language of the oWECS authors was English and the instrument was validated in English using conventional psychometric measures in English (9). The English oWECS assessment form consists of four behavioral subscales: Attraction, Vocal Communication, Facial Communication and Sensitivity-Reciprocity, along with a maximum of four very simple and user-friendly English descriptors to help the coder determine a score for each subscale domain (See Figure 1). Training on the oWECS included viewing a webinar in English and rating a set of training videos of English-speaking mother-child dyads. Trainees typically took a few weeks to achieve reliability and then advanced to using the oWECS in their research or clinical practice.

**Figure 2:**
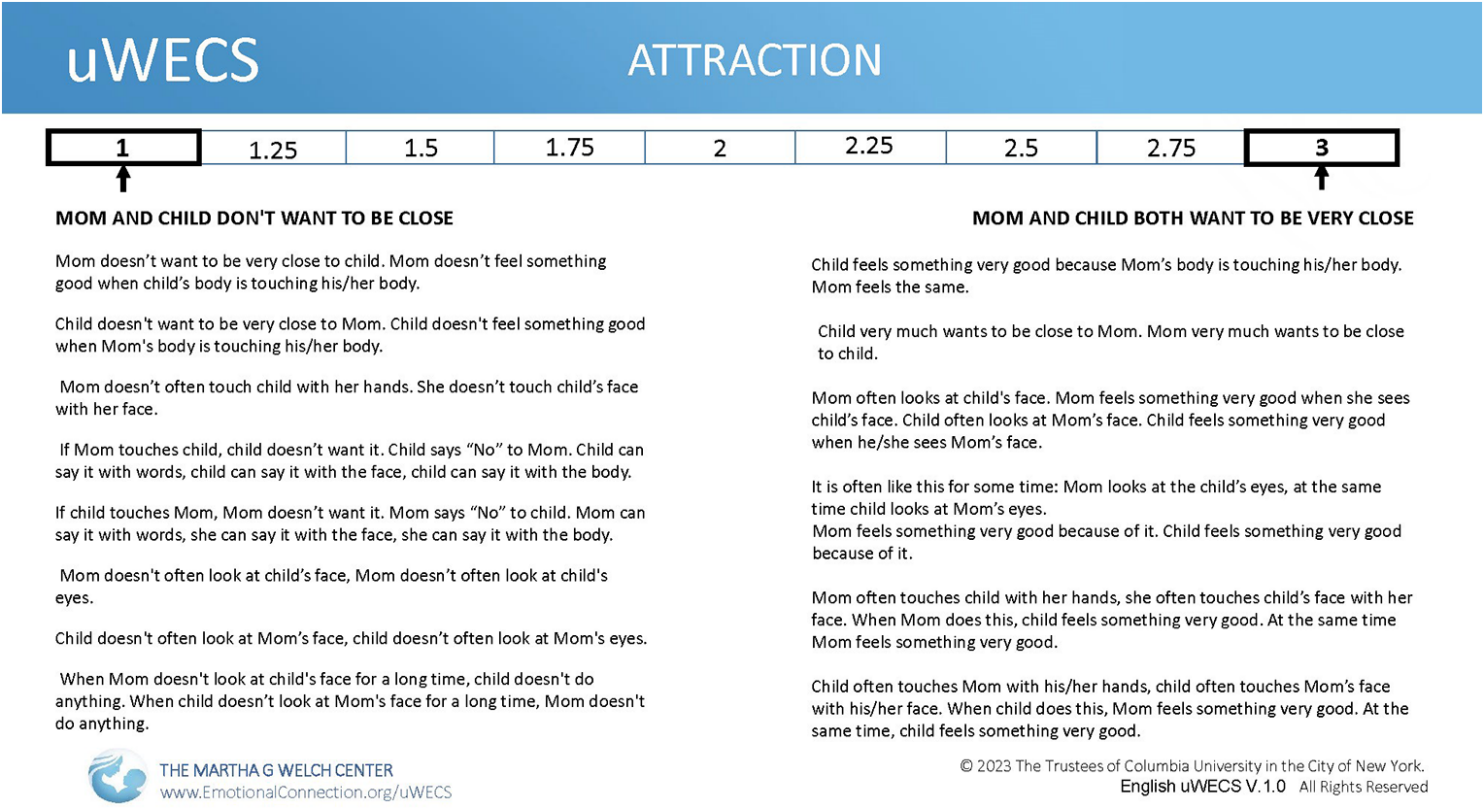
The English version of the universal welch emotional connection screen (uWECS) score sheet. Shown is the Attraction subscale with descriptors in clear, explicit translatable language. The idea behind the universal assessment tool is that the descriptors can easily be translated into any language and that by themselves the descriptors provide sufficient information to the observer that no additional training is necessary.

In the course of oWECS trainings and usage, it turned out that the simple English descriptors on the oWECS form led to interpretive and semantic confusion for both native and non-native English speakers, as well as for coders with different levels of education. A collaboration between author Welch and authors Vanhatalo and Goddard, who are experts in the field of natural semantic metalanguage (10, 11) led to creation of a version of the oWECS that was linguistically accessible to users across cultures, the Universal WECS (uWECS) (See Figure 2) (8). In so doing, the team utilized ∼65 so-called semantic primes, words that can be translated into any known language and retain their semantic representation, and that are unable to be defined using simpler terms. For example, while the term “mutual gaze” is not directly translatable into every language. Hence mutual gaze in the oWECS is written as “mother often looks at child's face; at the same time child looks at the mother's face” in uWECS.

Recently, in partnership with the Children's Learning Centers of Fairfield County (CLC), we completed a randomized controlled trial (RCT) of Mother-Child Emotional Preparation intervention (MCEP) (ClinicalTrials.gov Identifier: NCT03442439). CLC is a leading community-based pre-school education program serving ∼1,000 diverse families annually in Stamford, CT. The trial compared a group of children receiving CLC's standard curriculum with a group receiving standard curriculum plus MCEP. The school teaches emotional literacy (i.e., seeks to increase children's “emotional intelligence”) through the Yale RULER curriculum, with an emphasis on English and Spanish. However, reaching all students is a challenge, since CLC students come from as many as 30 different countries. MCEP is different from the RULER program in that it aims to increase the child's regulatory capacity by fostering AEC *with the parent*, as opposed to emotional intelligence of the child. The primary outcome of the RCT was AEC, as measured by the oWECS. In a separate publication (see Welch et al. in this special collection (12), we report that MCEP significantly improved AEC, as well as behavior regulation in the classroom and in the home.

In this paper, we use data from the MCEP RCT to validate the uWECS. We used videotaped interactions between mother-child subjects who were primary Spanish speaking to code the uWECS and who were blinded to the coding of the oWECS, which was coded by bilingual, primary English/secondary Spanish speaking coders. In doing so, we sought to demonstrate that the Spanish translation of the uWECS, when coded by Primary Spanish-Speaking coders, would yield results similar to the oWECS, when coded by coders who had access to much more elaborate behavioral descriptors written in English.

We had two aims: (1) to demonstrate *concurrent validity* of the Spanish uWECS, by having primary Spanish speaking coders (Spanish^P^) code the Spanish uWECS, compared with original oWECS coding by bilingual English^P^/^S^Spanish coders as presented in the MCEP efficacy study (12) and; (2) to demonstrate *criterion-related validity* by examining whether the uWECS ratings performed similarity to the oWECS ratings in evaluating the efficacy of the MCEP for only those dyads who spoke Spanish throughout the study (i.e., in the intervention sessions and in the observations filmed for research purposes). We hypothesized that the Spanish uWECS would: (1) achieve concurrent validity with the oWECS and (2) demonstrate criterion-related validity with oWECS vis-à-vis similar demonstration of the efficacy of the MCEP program.

## Methods

### Mother-child emotion preparation (MCEP) trial

Mother-Child Emotion Preparation (MCEP) is a preschool mother-child intervention aimed at improving mutual emotion expression and autonomic emotional connection (AEC) between mother and child in a culturally and linguistically diverse sample. MCEP is a *culturally competent* program, in the sense that it encourages mothers to communicate when possible in the mother's primary language (e.g., the language spoken to the mother when she was a child). We conducted a randomized-controlled trial of MCEP together with Children's Learning Centers of Fairfield County in Stamford, CT, (CLC) a community-based preschool education center. Mothers signed a consent form at the time of the baseline assessment and randomized to either a standard CLC curriculum group (SC) or a SC plus MCEP intervention group (MCEP). See Welch et al. (12), for a full description of the MCEP study.

Mother-child dyads randomized to the MCEP group received facilitated MCEP calming sessions in a group setting (4 to 8 mother-child pairs per group). Two specially trained *Nurture Specialists (NSs)*, both licensed clinical social workers, facilitated the parent-child intervention and provided emotional support during sessions. Each mother-child dyad participated in two to eight two-hour group sessions over sixteen weeks, which took place in a classroom or small meeting room at CLC. Calming sessions consisted of a child sitting on the mother's lap and cycling through a range of verbal and non-verbal emotional expression. This communication typically dealt with current or past upsets or other previously unprocessed feelings. A successful calming session included mother and child expressing a full range of emotions, including discomfort and distress, conflict and upset, conflict resolution and mutual calm. See (12) for a full description of the MCEP sample and procedure.

The efficacy of the MCEP across the full RCT sample has been demonstrated and includes increased mother-child emotional connection, reduced behavior problems as per teacher and parent report (12) and healthier autonomic responding to stress in children (13).

### Subjects

Children were eligible for the study if they were between 2 and 4.5 years of age at the recruitment date. In addition, the child had to be a singleton without genetic or congenital disorder or motor disability. Mothers had to be at least 18 years old, able to speak, read and write in English or Spanish; and be living with her child full-time. Mothers were excluded for severe mental illness or any other medical conditions preventing play activities; involvement with the Department of Children and Families; struggling with drug or alcohol abuse; pregnancy (second or third trimester) that could interfere with the lap-based procedures (see below); and if they were unable to commit to the study schedule.

More than half of families enrolled in the MCEP trial identified as Latinx, first-generation immigrants who spoke Spanish at home. This subgroup was bilingual, with Spanish being the primary language spoken and English being a second language.

From the subjects in the trial, we selected the sub-cohort of 52 primary Spanish-speaking dyads (See Table 1). There were two language groups at the time of enrollment, one whose primary language was Spanish and one whose primary speaking language was English. The two groups do not differ significantly from one another on these demographics (all *p*-values > .10). While 50% of the English-speaking subsample identified as Hispanic, those mothers reported English as their primary language spoken at home and hence were not in the sub-cohort used for the present study. Spanish-speaking mothers identified as Hispanic, and the large majority did not report their race, perhaps due to the lack of breadth in the demographic categories provided. Due to Covid-related school closures, there was a higher than anticipated attrition at the 6-month follow-up visit (See (12). Of the 52 Spanish-speaking families at the time of enrollment, 31 returned, representing 40.4% attrition from school closure. Those who did not return did not significantly differ from those who did, with all *p*-values on key demographics >.10.

**Table 1:** Demographics of participants at time of enrollment.

	Primary Spanish speaking	Primary English speaking
	*n* = 52	*n* = 38
Age at enrollment	Mean ± SD	Mean ± SD
Mothers’ age (years)	33.65 (4.79)	35.11 (5.24)
Child	3.9 (.39)	3.65 (.51)
Sex of child		
Male	26 (50.0)	23 (60.5)
Female	26 (50.0)	15 (39.5)
** **	*n* (%)	*n* (%)
Ethnicity of mother		
Hispanic	52 (100)	19 (50)
Non-Hispanic	0	19 (50)
Race of mother		
White	9 (17.3)	11 (28.9)
Black	0	7 (18.4)
Asian	0	4 (10.5)
Hawaiian/Pacific Islander	0	1 (2.6)
Indigenous	0	1 (2.6)
Other/not Reported	43 (82.7)	14 (36.8)
Mother relationship status		
Single	8 (15.4)	11 (28.9)
Married	23 (44.2)	22 (57.9)
Partner/Cohabitating	12 (23.1)	1 (2.6)
Partner/living Apart	3 (5.8)	0
Separated	5 (9.6)	2 (5.3)
Divorced	0	2 (5.3)
Not Reported	1 (1.9)	0
Mothers’ education		
Some schooling	14 (26.9)	7 (18.4)
High school or GED	18 (34.6)	13 (34.2)
Some college or associate's	11 (21.2)	6 (15.8)
Bachelor's and/or graduate degree	8 (15.4)	10 (26.4)
Unknown	1 (1.9)	2 (5.3)
Mothers’ employment		
Employed	30 (57.7)	20 (52.6)
Unemployed	21 (40.4)	17 (44.7)
Unknown	1 (1.9)	2 (5.3)
State/federal assistance		
Receive assistance	28 (53.8)	20 (52.6)
Do not receive assistance	18 (34.6)	14 (39.5)
Unknown	6 (11.5)	3 (7.9)

#### Assessment tools

##### Original welch emotional connection screen [oWECS; (9)]

**Figure 3:**
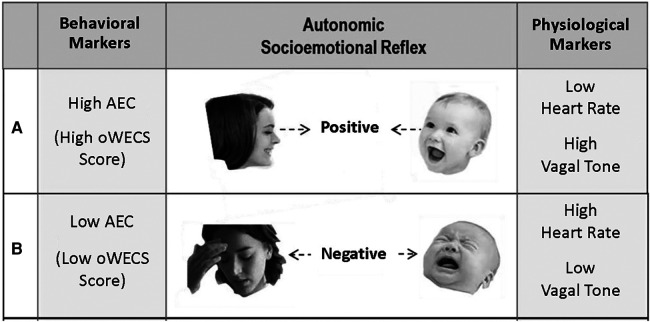
Schematic showing the opposite behavioral and physiological markers associated with the autonomic socioemotional reflex. Note that the hypothesized correlation between autonomic emotional connection (AEC) markers as assessed by the oWECS behavioral scores and physiological markers as assessed by heart rate and vagal tone.

The primary outcome for the MCEP trial was *autonomic emotional connection* (AEC), as assessed on the original *Welch Emotional Connection Screen (oWECS)* (9). The Lap-Check test is designed to capture the behavioral indicators of the AEC construct (see Figure 3), including the concomitant physiology (13), both of which are associated with the mother and infant/child *autonomic socioemotional reflex* (*ASR*) (14).

The oWECS rates mother-child AEC on four modality subscales: mutual *Attraction*, *Vocal Communication*, *Facial Communication, Mutual Sensitivity and Reciprocity* (see Figure 2). Each WECS dimension is rated on a 9-point scale, with scale anchors tied to behavioral descriptions at 1 (low), 2 (variable/mixed), and 3 (high). Scale ranges include.25 increments between each anchor (i.e., **1**, 1.25, 1.5, 1.75, **2**, 2.25, 2.5, 2.75, **3**). A high score on each dimension indicates that the dyad is emotionally connected via this modality. For this study, these four-dimensional WECS scores were averaged to create a composite AEC score that aligns with this scaling for interpretation—e.g., an oWECS score of 1–1.5 is low; 1.75–2.25 mid-range; and 2.5–3 high on AEC.

Mother-child AEC was assessed at baseline (i.e., enrollment) and 6-months following enrollment during a brief (5 min) emotional exchange known as the *Welch Orienting Lap Check* (12). During the Lap Check, mothers sat with their child on their lap in a face-to-face position and engaged with their child as they usually would for 5 min. There were no toys, food items, drinks, electronic devices, books or other objects of distraction in the room. Electrocardiogram (ECG) recordings were acquired from mother and child during this observation (for ECG results, see Myers et al., under review).

##### Universal welch emotional connection screen (uWECS)

The oWECS coding instrument was translated using Clear, Explicit, Translatable Language (CETL) (See Figure 3**)** to provide global access to the WECS and for any language speaker and for varying literacy levels of coders (8). In addition to increasing accessibility of measurement of AEC globally, the uWECS also opens the door for valid assessment of parent-infant/child interactions that are key to evaluating the efficacy of early relational health interventions in diverse samples such as was done with MCEP. CETL language is a derivative of Natural Semantic Metalanguage (NSM) (10, 15), or a lexicon of words identified by linguists to be translatable into any language globally (16).

For this study, the uWECS was translated into Spanish by a NAATI (National Accreditation Authority for Translators and Interpreters: https://www.naati.com.au/) certified translator linguist, who we used to ensure the highest professional translation standard. Of note, the professional translation did not differ from the translation completed by a Primary Spanish-speaking social worker and a Spanish-speaking undergraduate research assistant. In all three cases the translation of uWECS was additionally verified by back-translation with no words lost/altered. Professionally translated uWECS in Arabic, Chinese (Mandarin), English, Finnish, German, Italian, Polish, Russian, Spanish, Turkish are available for download at www.emotionalconnection.org.

### Statistical approach

This study had two aims. The first aim was to examine the concurrent validity of the Spanish uWECS. We hypothesized that the scores would be highly correlated. To test this hypothesis, we computed intraclass correlations (ICC); vs. Simple Pearson correlations. The ICC is calculated by determining both the linear trend and the similarity in mean values of the ratings of coders. The ICC is hence more conservative and appropriate for determining the reliability of observational coding with continuous/scaled measurement. Our second aim was to examine the criterion-related validity of the uWECS. We computed two ANOVA's to assess the significance of the MCEP and control groups on the oWECS and the uWECS. We hypothesized that the overall AEC scores on Spanish uWECS in the current study would demonstrate efficacy of the MCEP RCT (12) similar to the oWECS scores.

### Coding

All videos were selected from the MCEP RCT on the basis of language spoken in the observations (see Welch et al., 2023 this edition) (See Table 2).

**Table 2:** Concurrent validity study coding details.

	Part 1 English oWECS (12)			Part 2 Spanish uWECS (www.emotionalconnection.org)		
	Lapcheck VIDEOS primary Spanish	Lapcheck VIDEOS primary English	CODERS primary English		Lapcheck VIDEOS primary Spanish	CODERS primary Spanish
Baseline	*n* = 52 (56%)	*n* = 39 (44%)	*n* = 91 (100%)	Baseline	*n* = 52 (56%)	*n* = 52 (100%)
6 months	*n* = 31 (46%)	*n* = 36 (54%)	*n* = 67 (100%)	6 months	*n* = 31 (46%)	*n* = 31 (100%)

MCEP was facilitated in Spanish and in English. At baseline, 56% of the dyads were primary Spanish speaking and were faciliated by primary Spanish speaking therapist. 42% of the dyads were primary English speaking facilitated in English. For the first part of our coding study, Spanish and English videos were coded by primary English speakers and English videos were coded by primary English speakers. For the second coding study, Spanish videos were coded by primary Spanish coders. (See Table 3 for pooled intraclass correlations).

In Part 1 of the concurrent validity study, coders using the English oWECS were trained to reliability by the first author, a primary English-speaking trainer, using a slide presentation and a practice set of videos of mother-child dyads with a wide range of AEC scores. A team of six coders achieved reliability on the training set of the English oWECS in 2–3 weeks. Those coders trained on the oWECS training set then went on to achieve interrater reliability on 20% of the full MCEP sample. This training and coding were completed remotely (i.e., over Zoom) in the Spring of 2020 and supervised by the first author.

In Part 2 of the concurrent validity study, two coders using the Spanish uWECS were given a simple introduction to the concept of autonomic emotional connection (AEC) by the first author. Coders then achieved reliability on the uWECS working independently with the uWECS-Spanish prompts. Training, reliability and coding on the uWECS was completed in 2024 at Williams College under the supervision of the first author.

In Part 1 of the concurrent validity study, the lap-check videos of primary English- speaking dyads were recorded in English and the Spanish speaking dyads were recorded in Spanish. All videos in Part 1 were coded by a team of six primary English-speaking coders, half of whom were bilingual and fluent in Spanish as a second language. oWECS Coders achieved reliability on 16 cases from the MCEP sample. The average intra-class correlation coefficients for each dimension of the WECS pooled across coders were as follows: Attraction = 0.93, Vocal = 0.93, Facial = 0.95, Sensitivity/Reciprocity = 0.94. Coding was then completed by the reliable coders, with no coding of the same case twice (i.e., if a coder A coded baseline, then coder B coded the 6-month follow-up for that dyad). All coders were blind to all other study data.

In Part 2 of the study, two primary Spanish speaking coders trained exclusively together on the Spanish uWECS and achieved interrater reliability across 16 cases coded independently and in duplicate. ICC ratings were as follows: Attraction: .879; Vocal .978, Facial: .950; Sensitivity/Reciprocity: .953. Coding was then completed by the reliable coders, with no coding of the same case twice (i.e., if a coder A coded baseline, then coder B coded the 6-month follow-up for that dyad). All coders were blind to all other study data.

## Results

### Aim 1: concurrent validity of the uWECS

We examined concurrent validity of the uWECS by comparing the subscales and overall AEC scores on the the English oWECS (See Table 3).

**Table 3:** Pooled intraclass correlations between the English oWECS and the Spanish uWECS scores at baseline and six months post-enrollment.

Subscales	Intraclass correlation coefficient (ICC)
Attraction	.81*
Vocal communication	.82*
Facial communication	.84*
Sensitivity/reciprocity	.81*
Average emotional connection score	.86*

oWECS, original welch emotional connection screen (9); uWECS, universal welch emotional connection screen (8); MCEP, mother-child emotional preparation program.

* *p* < .001.

Results showed that MCEP significantly improved AEC for primary Spanish speaking dyads when rated with the English oWECS *and* the Spanish uWECS. Results support the concurrent validity of the uWECS.

Note that Table 3 shows the intraclass correlations between English oWECS and Spanish uWECS pooled across the baseline and 6-month post-enrollment observations. The associations are robust, ranging from .81 to .84, with the composite average scores on both instruments achieving an ICC of.86. All values are significant at *p* < .001. Agreement between the ratings from blinded, independent teams of coders on the English oWECS and the Spanish uWECS demonstrate sound concurrent validity of the uWECS.

The validity of the uWECS opens the door for researchers across the globe to use the instrument for research or clinical practice without linguistic barriers for the coder.

### Aim 2: criterion-related validity of the uWECS

**Figure 4:**
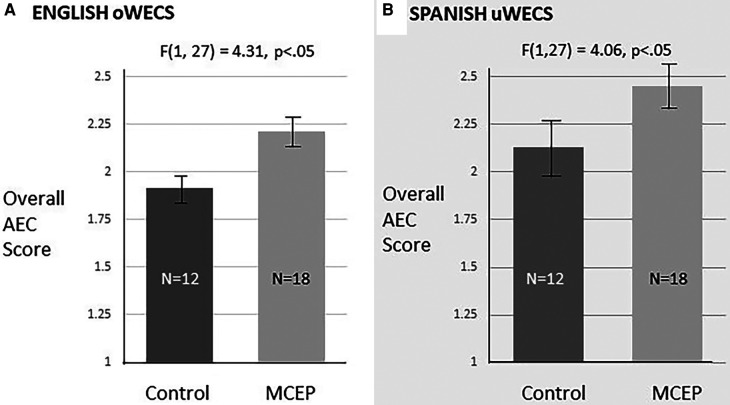
Graphs showing average overall autonomic emotional connection (AEC) scores on the English oWECS and spanish uWECS at 6 months post enrollment. (**A**) The English oWECS was coded by primary English speaking coders trained to reliability via a traditional method. (**B**) The Spanish uWECS was coded by primary Spanish speaking coders using only the uWECS form with little training. Note that both codings showed significant increase in overall emotional connection scores. The Spanish uWECS results were very similar to the English oWECS results. These results confirm Aim 2 of the study by establishing criterion-related validity of the uWECS. Both analyses controlled for baseline scores.

We examined criterion-related validity of the uWECS by comparing overall AEC scores on the English oWECS and Spanish uWECS. First, two ANOVAs were computed to examine the MCEP sub group and control group differences at baseline and six months post-enrollment on English oWECS and Spanish uWECS scores. As expected, given random assignment, there were no significant differences at baseline between scores. However, at 6-month follow-up, controlling for baseline scores, analyses of covariance (ANCOVA's) performed on the English oWECS and Spanish uWECS data revealed that the MCEP group showed the same significant improvement in AEC on both instruments: English oWECS, *F*(1, 27) = 4.31, *p *< .05, $\eta_{p}^{2}=.14$; and Spanish uWECS, *F*(1, 27) = 4.06, *p *< .05, $\eta_{p}^{2}=.13$ (Figure 4). These similar results in evaluation of the AEC construct on the two instruments support criterion-related validity of the uWECS.

## Discussion

In this study, we demonstrated both concurrent and criterion-related validity of the uWECS (8), a universally translatable version of the original Welch Emotional Connection Screen (oWECS) (9). Findings mark an important advance in the field of early intervention by demonstrating the value in applying assessment tools that work effectively and equally across cultures and languages to interventions that may (or may not) be competent across cultures and languages.

Results of this validation study indicate that nothing is “*lost in translation*” by minimizing grammatical complexity, word choice, and literacy level in the creation of the uWECS instrument. The findings of Jakael et al., (This issue under Review) further support that coders were able to achieve reliability in a multilingual online training session, as the uWECS was readily translatable live and in the training platform to both German and Finnish for a group of coders that included both bilingual (i.e., English/German or English/Finnish) and monolingual German or Finnish coders. The uWECS paves the way for clinicians and researchers around the globe to reliably rate AEC with a validated uWECS instrument.

In addition to validating the uWECS in another language, the demonstrated efficacy of MCEP for exclusively Spanish-speaking dyads underscores the cultural competence of the MCEP. We note that MCEP demonstrated sustained efficacy despite losing approximately 40% of the sample size due to Covid-related school closure. In addition, the efficacy of MCEP for Spanish as the primary language mothers and young children suggests that MCEP is a culturally-competent intervention for Latinx mothers and children and that it was possible to make accessible in the community. Given the vulnerability of Spanish children to be removed from preschool programming (16, 17) and the evidence that emotion understanding is a challenge for young children who are learning English as a second language, the sustained increase in emotional connection for mothers and children may promote emotional awareness and understanding that carries over to the classroom environment (12).

First- and second-generation immigrant mothers are under-served in seeking support (18) and prefer informal programs to support their children in their community (19). MCEP sought to reach immigrant mothers by creating an accessible program, provided in the trusted early childhood learning center, and with other primary Spanish speaking mothers, children as well as social worker. Building from a sense of community and linguistic affiliation may have provided the safe space for mothers’ expression of emotion with children, which in turn increased the likelihood that mothers would continue to tap into the MCEP curriculum tools after the program ended, thus explaining the sustained effects of MCEP on EC months after completing the intervention.

A separate report in this special topic journal provides first proof-of-concept that the quality of dyadic autonomic emotional connection among culturally and linguistically diverse infants and parents can be reliably assessed with the uWECS (see Jaekel et al. this issue under review). The study tested the use of the uWECS translated into German and Finnish in an online multilingual group training program. Results showed coders speaking diverse primary languages, including Russian, Turkish, Kurdish and Twi, achieved sound interrater reliability when coding videos.

Our sample of videos was collected in the school setting, with bilingual mothers and children working with a social worker who also spoke Spanish as their primary language. Emotion expression, which is the focal point of MCEP, was guided by the interventionist with all mothers encouraged to speak with their child in the language spoken at home. Minoritized, low income and preschoolers for whom English is a second language show more dysregulated behavior (20), placing them at-risk for loss of a key resource needed to succeed in future education. The findings here point to the value of a culturally competent assessment tool that can be used effectively in multiple languages to measure autonomic emotional connection between mother and child and in the school context. Our findings support the concurrent and criterion-related validity of the uWECS, one that can be reliably coded by coders who speak *any* language.

The uWECS offers an important tool for educators. American preschools currently lack a key resource for the children most in need of preschool programming, such as children for whom English is a second language (21). The uWECS is an assessment tool that is culturally sensitive and accurate. Emotional assessment tools and interventions are also lacking for first- and second-generation immigrant parents of young children (18, 22). Fear of deportation, discrimination, cultural incompetence, linguistic barriers and stigma deter help-seeking for Latinx immigrants (23). However recent evidence suggests that Latinx adults seeking selfcare and Latinx parents seeking help for their children report a preference to seek informal help from resources from those who speak the same language and share the same cultural experiences.

Young children are in need of tools to assess the relational health with parents to improve academic success in preschool, kindergarten and early elementary school (24). A recent study of bilingual 5–6-year-old children found that bilingual children identify emotions as well as monolingual children, but are not as adept at understanding the mental sources of emotion (i.e., desires, beliefs, memories or culture as influences of emotions) as monolingual children (25). Hence, cultural and linguistic barriers for young children may predispose them to manifesting dysregulated behavior because of a lack of understanding of the emotional states of those who do not share their language or cultural display rules.

The ‘autonomic’ emotional connection (AEC) is a novel construct that we created to describe the mutual behaviors of mother and baby or child that are triggered by the autonomic nervous system. AEC differs from “psychological” emotional connection construct that has been used in academic studies to describe the parent-child relationship since at least the 1990s (26). Behaviors triggered by autonomic state require special assessment in order to differentiate them from conscious and unconscious psychologically triggered behaviors. Conventionally validated assessment tools that are based on “attachment” and “bonding” theory and Psychological emotional connection constructs (27) often measure separate psychological behaviors of infant and mother but not the dyad's mutually Engaged behaviors.

### Limitations and future directions

This study was limited by the loss of participating dyads due to Covid-related school closure. Hence these findings are limited by a small sample size and the context of the Pandemic. As well, while the MCEP delivered the curriculum in the school setting by a primary Spanish speaking interventionist and with other primary Spanish speaking families, it is important to note that sharing a common language does not necessarily mean shared culture, immigration status, or life history. It is essssential to replicate this work on the Spanish u-WECS with other Spanish-speaking communities in and outside of the US. It is noteworthy that affiliation grouping by language for the MCEP, and use of the uWECS-Spanish demonstrated sustained effects for primary Spanish speaking families. This points to the clear benefit of the MCEP's approach to intervention, the sustainability of higher EC in Latinx dyads and the ability to validly measure emotional connection via a translated version of the uWECS coded by primary Spanish speaking coders. This study is limited by the singular transaltion of the uWECS into Spanish. It is important to assess the validity of the uWECS translated into other languages. Adapting and validating a universal WECS instrument paves the way for continued research in AEC globally, opening the door for continued understanding of this novel biobehavioral construct.

## Data Availability

The raw data supporting the conclusions of this article will be made available by the authors, without undue reservation.
